# Complementation of two related tumour cell classes during experimental metastasis tagged with different histochemical marker genes.

**DOI:** 10.1038/bjc.1993.170

**Published:** 1993-05

**Authors:** W. C. Lin, K. L. O'Connor, L. A. Culp

**Affiliations:** Department of Molecular Biology and Microbiology, Case Western Reserve University, School of Medicine, Cleveland, Ohio 44106.

## Abstract

**Images:**


*Br. J. Cancer* (1993), **67**, 910–921 © Macmillan Press Ltd., 1993

### Complementation of two related tumour cell classes during experimental metastasis tagged with different histochemical marker genes*

W.-C. Lin, K.L. O'Connor & L.A. Culp

*Department of Molecular Biology and Microbiology, Case Western Reserve University, School of Medicine, Cleveland, Ohio 44106, USA.*

**Summary** Intercellular complementation during tumour development and metastasis was analysed for two different oncogene (*ras* or *sis*) transformants of Balb/c 3T3 cells, tagged with different histochemical marker genes (*lacZ* or *ALP* to generate LZEJ or APSI cells, respectively), by localising them after their co-injection with specific double-staining protocols. This model evaluates whether limited progression of each tumour class can be facilitated reciprocally during co-localisation and co-growth in nude mice by taking advantage of the sensitivity of the histochemical marker genes for localising them. After intravenous co-injection of equal numbers of both cells to analyse experimental metastasis, most foci transiently established in the lung for several hours were comprised of only one cell class. However, a significant fraction of foci contained both cell types, as identified in double-stained whole-lung tissues and in lung sections. Evidence was obtained that LZEJ cells increase the survivability and subsequent growth of APSI-containing micrometastases during co-localisation in lung, when compared to APSI cells injected alone. Conversely, APSI cells facilitate expansion of LZEJ cells from micrometastatic foci into overt-metastatic nodules in the lung. These analyses reveal reciprocity during experimental metastasis by two related tumour cell classes derived from the same parental cell.

The *ras* oncogene conveys some metastatic competence to tumour cells in a large number of biological systems (Barbacid, 1987; Egan *et al.*, 1987; Fidler & Balch, 1987; Hart *et al.*, 1989; Hart & Saini, 1992; Miller & Heppner, 1990; Nicolson, 1988). In previous studies from this laboratory (Radinsky & Culp, 1991; Radinsky *et al.*, 1987), it was shown that Kirsten murine sarcoma virus-transformed Balb/c 3T3 cells could form pulmonary micrometastases in athymic nude mice but overt metastases could be detected rarely, with the clonal dominance of very select cell types within the *Ki-ras*-transformed cell population during tumour progression (Radinsky & Culp, 1991). These results suggested that the primary effect of *ras* in a Balb/c 3T3 recipient was the promotion of escape of selected cells from the primary tumour site and their subsequent invasion of a target organ but not efficient outgrowth, indicating again that other growth-promoting genes are required for complete metastatic expansion. These requirements might include any host organ-specific growth factor (Cavanaugh & Nicholson, 1989), as well as growth factors supplied by the tumour cells themselves (Goustin *et al.*, 1986; Hart *et al.*, 1989; Hart & Saini, 1992). In the experiments reported here, we test a model for the paradigm in which two different tumour cell classes, highly related to each other genetically but expressing different genes potentially important for complete metastasis, arise during tumour development from the same parental cell. These two classes of tumour cells would then facilitate progression for each other at discrete stages of metastasis.

In order to follow the fates of human EJ-*ras*-transformed Balb/c 3T3 cells more effectively *in situ* both qualitatively and quantitatively, we used the bacterial *lacZ* marker gene to tag *ras*-transformed 3T3 (LZEJ**, abbreviation for LacZ/EJ-Ha-*ras*-expressing cells), permitting their detection as blue-staining single cells minutes-to-weeks after injection (Lin *et al.*, 1990*a*). Ultrasensitive detection of the *lacZ* marker gene enables us to follow the earliest stages of development of micrometastatic foci at target organs and to quantitate the metastatic potential of LZEJ cells more accurately (Lin *et al.*, 1990*a*,*b*). The micrometastasis pattern in this LZEJ system with plasmid-transfected EJ-Ha-*ras* oncogene is very similar to that observed in the viral Kirsten-*ras* transformation system, suggesting again that the *ras* oncogene may effect stable invasion of the lung by a small subset of 'transformed' Balb/c 3T3 cells and that only a very small subset of these become successful at forming overt metastases (Lin & Culp, 1992*a*; Lin *et al.*, 1990*a*,*b*). Therefore, growth and population expansion of micrometastatic cells at target organs are limited, except in a few exceptional cases.

Heterogeneity of tumour cell subpopulations and their cooperation in contributing to malignant tumour progression have been studied in only a few systems (Aslakson *et al.*, 1991; Hart & Saini, 1992; Heppner & Miller, 1983; Maghazachi *et al.*, 1988; Miller *et al.*, 1980; Miller & Heppner, 1990). Interactions among subpopulations of tumour cells could very well determine the outcome of metastasis (Hart *et al.*, 1989; Miller & Heppner, 1990). To investigate the requirements for maximising/minimising tumour progression in the studies described herein, parental Balb/c 3T3 cells (not harbouring the *ras* oncogene) were transfected with another oncogene whose product may complement the activities of *ras*-transformed 3T3 cells by intercellular mechanisms – the *sis* oncogene, whose PDGF growth factor product (PDGF B-chain) stimulates growth of 3T3 cells and their derivatives. *Sis* has been shown competent for transforming Balb/c 3T3 cells (Sugita *et al.*, 1992; Westermark & Heldin, 1991; Zhan & Goldfarb, 1986). Since proliferation of parental and derivative Balb/c 3T3 cells is dependent on exogeneous PDGF, PDGF production by a second cell class may facilitate growth of *ras*-transformed micrometastatic cells in the lung and facilitate tumour progression. Conversely, *ras*-transformed cells may provide factors or microenvironments that facilitate progression of the *sis* transformed cells. To analyse these possibilities, human c-*sis*-transfected 3T3 cells were tagged with a second histochemical marker gene (human placental alkaline phosphatase [Lin & Culp, 1991]) in order to examine the 'trans' complementation of two related tumour cell types, one containing *ras* and the other *sis*.

### Materials and methods

The following experimental materials were obtained from commercial sources: tissue culture flasks and multiple-well

Correspondence: L.A. Culp.

*Presented at the 33rd meeting of the British Association for Cancer Research, April 1992, Southampton, UK.

**The abbreviations used are *lacZ*, *Escherichia* β-galactosidase gene; X-gal, 5-bromo-4-chloro-3-indoyl-β-D-galactopyranoside; LZEJ, *lacZ* and EJ Ha-*ras* transfected Balb/c 3T3 cells; X-phosphate, 5-bromo-4-chloro-3-indoyl phosphate; APSI, alkaline phosphatase and human c-*sis* transfected Balb/c 3T3 cells.

Received 8 September 1992; and in revised form 2 December 1992.

dishes from Becton Dickinson Labware, Oxnard, CA; neonatal calf serum from Biologos Co., Naperville, IL; Permount, acetone, formaldehyde, and microscope slides from Fisher Scientific Co., Fairlawn, NJ; Dulbecco's modified Eagle's medium (DMEM) and G418 sulfate from Gibco Co., Grand Island, NY; glutaraldehyde from Eastman Kodak Co., Rochester, NY; glycol methacrylate embedding kit (JB-4) from Polysciences, Inc., Warrington, PA; HEPES, RNase A, naphthol AS-BI phosphate, fast red TR, neutral red, methyl green, potassium ferricyanide, potassium ferrocyanide, and paraformaldehyde from Sigma Chemical Co., St. Louis, MO; 5-bromo-4-chloro-3-indoyl-β-D-galactopyranoside (X-gal), 6-chloro-3-indoyl-β-D-galactopyranoside (Red-gal), 5-bromo-4-chloro-3-indoyl phosphate (X-phosphate), p-nitro blue tetrazolium chloride (NBT) from Research Organics, Cleveland, OH.

*Cell culture and transfection*

All cell lines were free of *Mycoplasma* and grown in Dulbecco's modified Eagle's medium with 250 units $ml^{-1}$ penicillin, 250 $\mu g\ ml^{-1}$ streptomycin sulfate, and 10% newborn bovine serum. The generation of the tumour-progressing LZEJ clone (abbreviation for LacZ- and human EJ-Ha-*ras*-expressing cells) by transfection of Balb/c 3T3 (clone A31) cells, as well as the genetics and expressions of the human EJ-Ha-*ras*, neomycin$^R$, and *lacZ* genes in this clone after transfection, have been described previously (Lin *et al.*, 1990*a*). Marker gene expression was stable during one round of tumour progression in nude mice, but was frequently lost during a second round of passage of tumour cell populations (Lin *et al.*, 1990*a*; Lin & Culp, 1992*a*).

To generate *sis* oncogene-transformed cells containing a second histochemical marker gene, the pRSVPAP and pREP-Isis plasmids were transfected into Balb/c 3T3 (clone A31) cells by the calcium phosphate precipitation technique. The human tumour origin and activity of this c-*sis* gene, coding for the B chain of PDGF, have been described previously (Beckmann *et al.*, 1988), including its ability to transform 3T3 cells (Sugita *et al.*, 1992; Zhan & Goldfarb, 1986). The generation and activity of the pRSVPAP plasmid in 3T3 cells has also been described (Lin & Culp, 1991). This plasmid expresses the human placental alkaline phosphatase (ALP) gene using several different substrates to generate different coloured products in transfected cells (Lin *et al.*, 1992). After G418 selection of transfectants and their outgrowth in soft agar, two clones were selected for these studies based on excellent histochemical staining – clone APSI (abbreviation for Alkaline Phosphatase- and SI*s*-expressing cell) and clone APB. APSI cells express high levels of human c-*sis* mRNA, as determined on northern blots, while APB cells express amounts of c-*sis* mRNA that are below detection by northern analysis; both clones stain intensely when assayed for human ALP activity (Lin *et al.*, 1992).

*Animals and tumour cell inoculation*

Animals were maintained pathogen-free in the Athymic Animal Facility (Case Western Reserve University Cancer Center). For individual injections, LZEJ or APSI cells ($1 \times 10^5$ cells/0.2 ml PBS suspended to guarantee single cells as verified by plating cells into culture dishes) were inoculated intravenously into 6 to 8 week old female athymic nude mice (HSD nu/nu). After sacrifice of animals at the indicated times, lungs were removed for evaluation of tumour cell distribution by histochemical staining as specified below.

*β-Galactosidase staining*

Tissue cultured cells were rinsed with PBS and fixed for 5 min at 4°C with 2% (v/v) formaldehyde, 0.2% (v/v) glutaraldehyde in PBS. The fixed cells were rinsed with PBS three times and then incubated at 37°C overnight in stain solution containing 1 mg $ml^{-1}$ X-gal to stain cells blue (or Red-gal as indicated to stain cells red (Lin *et al.*, 1992)), 20 mM potassium ferricyanide, 20 mM potassium ferrocyanide, and 2 mM $MgCl_2$ in PBS. For whole-organ staining, selected organs were removed from animals immediately after sacrifice. After fixation in 2% (v/v) formaldehyde, 0.2% (v/v) glutaraldehyde in PBS for 60 min, these organs were rinsed with PBS three times and incubated in the X-gal staining solution. Nonidet-P40 and sodium deoxycholate were added to the stain solution to final concentrations of 0.02% (v/v) and 0.01% (w/v), respectively. After incubation at 4°C overnight, the tissues were rinsed briefly with 3% (v/v) dimethyl sulfoxide in PBS and then with PBS (Lin *et al.*, 1990*a*). Samples were stored at 4°C in 0.02% (w/v) sodium azide in PBS before microscopic evaluation and photography.

*Alkaline phosphatase staining*

Tumour cells bearing the human ALP marker gene were detected by histochemical staining in order to differentiate them from *lacZ*-bearing tumour cells (Lin *et al.*, 1992). Cultured APSI cells were fixed for 5 min at 4°C with 2% (v/v) formaldehyde, 0.2% (v/v) glutaraldehyde in PBS, rinsed with PBS three times, and incubated at 37°C or 4°C for 30 min with 1 mg $ml^{-1}$ X-phosphate, 1 mg $ml^{-1}$ NBT in pH 10.0, 0.1 M Tris buffer to stain cells reddish-black; to stain them blue, cells were treated with X-phosphate *without NBT* (Lin *et al.*, 1992). Cells were rinsed with PBS and stored at 4°C in 0.02% (w/v) sodium azide in PBS.

For whole-organ staining to detect ALP activity, tissues were rinsed with PBS, fixed for 60 min at 4°C with 2% (v/v) formaldehyde, 0.2% (v/v) glutaraldehyde in PBS, heated at 65°C for 30 min, rinsed with PBS three times, and incubated at 4°C overnight with 1 mg $ml^{-1}$ X-phosphate, 1 mg $ml^{-1}$ NBT, 0.02% NP-40, 0.01% sodium deoxycholate in 0.1 M Tris buffer pH 10.0 to stain tumour cells reddish-black (alternatively, with X-phosphate without NBT to stain them blue). The 65°C heating step is critical to inactivate any host tissue and blood vessel alkaline phosphatase activity, while conserving the activity of the transfected placental form of alkaline phosphatase which is resistant to this heat treatment (Henthorn *et al.*, 1988). After staining, tissues were rinsed with PBS and stored at 4°C in 0.02% (w/v) sodium azide in PBS.

*Analysis of tissue sections*

To evaluate in some detail the locations of the two tumour cell classes, tissues previously stained for *lacZ* and/or for ALP gene activities (described above) were cut into 4-μm thick sections after embedding in methacrylate as described previously (Lin *et al.*, 1990*a*). They were also counterstained with neutral red in order to improve resolution of the host cells with the two tumour cell classes.

*Photomicrography*

Photomicrographs of sections and cultured cells were obtained with a Nikon Diaphot-TMD microscope equipped with a Microflex AFX. Photomicrographs of whole organs were obtained with a Nikon SMZU dissecting microscope equipped with a Microflex UFX.

### Results

*Double-staining analyses of mixed cell populations*

Double-staining protocols are used to resolve two tumour cell classes tagged with different histochemical marker genes (Lin & Culp, 1991; Lin *et al.*, 1992). The naphthol-ASBI (or naphthol-ASMX)/fast red protocols for alkaline phosphatase activity are unsuitable for whole-organ staining to detect ALP-tagged tumour cells because of high background staining of blood vessels and some organ cells. The X-phosphate/NBT protocol described in this study is far more sensitive for cell detection and can be used for whole-organ staining upon heat treatment to reduce background staining. This results in

**Figure 1** Short-term fate of APSI cells in the lung after i.v. injection. APSI tumour cells were detected after staining of the whole lung with the X-phosphate/NBT combination. **a**, A lung from a mouse given an i.v. injection of $1 \times 10^5$ APSI cells, 1 h post-injection. Reddish-black staining microfoci are numerous, a few of which are indicated by white arrowheads (Magnification, 20 ×). **b**, A lung from a mouse at higher magnification, 1 h post-injection. Blood vessel structures, indicated by black arrows, are apparent because of the persistence of red blood cells (Magnification, 100 ×). Most microfoci become localised at the ends of the smallest blood vessels in the lung. **c**, A lung from a mouse at low magnification, 24 h post-injection. Reddish-black staining APSI microfoci are far fewer in number than at early time points (compare with **a**) and are indicated by white arrowheads (Magnification, 20 ×).

Figure 2

**Table I** Quantitation of pulmonary micrometastases/nodules[a]

| | *APSI injected singly* | | | *APSI Co-injected with LZEJ* | | | | |
|---|---|---|---|---|---|---|---|---|
| *Time of sacrifice* | *Micro-metastases*[b] | *Staining nodules*[c] | *Non-staining nodules*[c] | *Double staining foci*[d] | *LZEJ nodules*[c] | *APSI nodules*[c] | *non-staining nodules*[c] | *double staining nodules*[c] |
| 1 h | 2,500–3,000 (100) | 0 | 0 | 500 (100) | 0 | 0 | 0 | 0 |
| 6 h | 700 (28–23) | 0 | 0 | 122 (24) | 0 | 0 | 0 | 0 |
| 24 h | 104 (4.2–3.5) | 0 | 0 | 24 (5) | 0 | 0 | 0 | 0 |
| 3 weeks | 38 (1.5–1.3) | 10 | 0 | 3 (0.6) | 26 | 20 | 7 | 3 |
| 5 weeks | 37 (1.5–1.2) | 54 | 17 | ND[e] | ND[e] | ND[e] | ND[e] | ND[e] |
| 7 weeks | 8 (0.3) | 10 | 5 | ND[e] | ND[e] | ND[e] | ND[e] | ND[e] |

[a]Mice (24 for two separate experiments) were given i.v. injections of $1 \times 10^5$ APSI cells alone or as a mixture with $1 \times 10^5$ LZEJ cells as indicated. At various times post-injection, mice were sacrificed; whole lungs removed, rinsed with PBS and stained with X-phosphate (or sequentially with X-gal and then with X-phosphate/NBT in the case of co-injections of LZEJ and APSI cells). These values for $1 \times 10^5$ LZEJ cells injected alone have been published previously (Lin *et al.*, 1990*b*). [b]Values = Number of micrometastases determined with the use of a dissecting microscope. Values in parentheses represent the number of foci remaining in the lung as a per cent of the 1 h value. [c]Denotes nodules of considerable size (>100 cells). Nodules which were heterogeneous in their staining for the histochemical marker genes are referred to as non-staining nodules. [d]These are the number of micrometastases containing both LZEJ and APSI cells in co-localised foci. Values in parentheses represent the number of foci remaining in the lung as a per cent of the 1 h value. The maximal number of foci of all cell classes (LZEJ-only plus APSI-only plus co-localised foci) observed at any time point was 6–7,000. [e]Not determined.

intense reddish-black staining of APSI cells in culture or organs; alternatively, use of X-phosphate alone (without NBT) results in blue staining of APSI cells (Lin *et al.*, 1992). It should be noted that X-gal (or Red-gal) treatment of APSI cells failed to stain them at all; similarly, X-phosphate (± NBT co-substrate) treatment of LZEJ cells failed to stain them (data not shown), demonstrating specificity of the histochemical substrate for the respective marker gene-coded enzymes (Lin *et al.*, 1992). For mixtures of LZEJ and APSI cells *in vitro* or *in vivo*, it is critical that the X-gal (or Red-gal) staining be performed first because of the heat sensitivity of the bacterial enzyme; cells or tissues can then be heat-treated at 65°C to eliminate host tissue alkaline phosphatase activity for subsequent staining with X-phosphate ± NBT to identify APSI tumour cells.

### *Tumour progression of APSI cells*

It was first essential to define the tumorigenic and metastatic potential of the APSI cells (in the absence of any other cell type) when they were injected into nude mice. The latency of APSI primary tumour development at subcutaneous sites is approximately 20 days – longer than LZEJ primary tumour latency (14 days). Of note, no micrometastases were ever observed in the lungs of animals when APSI cells were injected s.c., while micrometastases of LZEJ cells were readily detectable after s.c. injection (Lin *et al.*, 1990*a,b*). Therefore, primary tumorigenicity and metastatic potential of *sis*-transformed Balb/c 3T3 cells are much poorer than those of *ras*-transformed 3T3 cells.

The fate of APSI cells in the lungs after i.v. injection was explored for comparison with LZEJ cells reported previously (Lin *et al.*, 1990*b*). Kinetics of clearance of APSI cells within 24 h are slightly different from those of LZEJ cells. As shown in Figure 1a and Table I, APSI cells populated the lung in large numbers during the first several hours, numbers similar to those observed with LZEJ cells (Lin *et al.*, 1990*b*). At higher magnification (Figure 1b), it becomes apparent that these APSI microfoci have established themselves at the ends of very small blood vessels identified by persistence of hemoglobin-containing cells and by their branching networks. Clearance of most APSI microfoci from the lung has occurred by 6 h (Table I), a rate which differs from LZEJ clearance where the number of microfoci remains high from 5 min to >6 h (Lin *et al.*, 1990*b*). APSI focus clearance continues between 6 and 24 h when a baseline of foci become established (white arrowheads in Figure 1c). Some of these persist for several weeks as micrometastases and some grow into sizeable metastatic nodules (Table I).

Complexity of clonal evolution of APSI tumour cell variants during experimental metastasis is documented more fully in Figure 2. Five weeks after i.v. injection, most APSI microfoci persist as small micrometastases with uniform staining for the ALP marker gene (Figure 2a and b). As a few nodules develop (Figure 2c), it becomes apparent that some intense-staining APSI cells grow clonally in localised regions of the nodule while $ALP^-$ (i.e. non-expressing) variants are generated in other localised regions. This is more evident in larger nodules (Figure 2d, e and f) where $ALP^-$ variants overgrow $ALP^+$ cells and become the dominant cell type in some nodules. Since APSI cells were cloned and were homogeneously staining for the ALP marker gene during their *in vitro* history, these results indicate that $ALP^-$ variants arise during cell division processes *in vivo* and that some of these variants may have selective advantage in forming larger lung nodules for any one of a number of reasons.

### *Co-development of APSI and/or LZEJ foci during experimental metastasis*

LZEJ and APSI cells were then followed after their co-injection i.v. to evaluate any cooperative interaction between these two related tumour cell types during establishment clearance, and nodule development in the lung. Two different

**Figure 2** Staining for alkaline phosphatase activity in lungs to detect experimental micro/metastases at later time points. Whole lungs from animals given an i.v. injection of $1 \times 10^5$ APSI cells were fixed and stained at the indicated time points (later than those of Figure 1) for ALP gene activity using the X-phosphate/NBT combination. **a**, Reddish-black-staining micrometastatic foci, whose size and morphology compare with those at the earliest time points, can still be identified at 5 weeks post-injection (Magnification, 100 ×). **b**, A small micrometastatic focus of stainable tumour cells at the edge of the lung where a larger metastatic nodule is developing, 5 weeks post-injection (Magnification, 100 ×). **c**, A heterogeneous reddish-black staining small nodule is identified, 5 weeks post-injection (Magnification 100 ×). This nodule is larger than the metastases shown in **a** and **b** and contains several intensely staining foci of expanding cell numbers, as well as neighbouring regions of tumour cells where stainability is much weaker. **d**, A large nodule observed at 3 weeks post-injection illustrates the increasing heterogeneity of tumour cell staining as the metastatic nodule develops (Magnification, 100 ×). There are many intensely staining foci of tumour cells concentrated at the periphery of the nodule and many non-staining regions which concentrate in the interior of the nodule. **e**, At lower magnification, a large heterogeneous reddish-black staining nodule is identified, 5 weeks post-injection (Magnification, 45 ×). **f**, Higher magnification of panel **d** (Magnification, 100 ×). This illustrates the heterogeneity of $ALP^+$ and $ALP^-$ foci that contribute to the expanding metastatic nodule in the lung.

**Figure 3** Distribution of APSI and LZEJ tumour cells in the lung soon after their intravenous co-injection. Lungs were removed from mice given an i.v. injection of a mixture of $1 \times 10^5$ LZEJ cells and $1 \times 10^5$ APSI cells, 1 h post-injection. Lung were fixed and stained with Red-gal substrate first; then heat-treated; and finally stained with X-phosphate alone. Red-stained LZEJ foci, well-isolated from APSI foci, are indicated by black arrowheads; blue-stained APSI foci, well-isolated from LZEJ foci, are indicated by black arrows. Two double-stained foci, containing both tumour cell types, are also observed at a significant frequency and are indicated by open arrows. **a** 100 ×. **b**, 200 ×. **c**, 300 ×.

double-staining regimens – X-gal and X-phosphate/NBT (or Red-gal and X-phosphate) – were used for maximising resolution of the two tumour cell classes in common environments, depending upon whether sections or whole organs respectively were being analysed. In all cases, the two cell types were mixed thoroughly in a tube prior to injection and for standardisation to the experiments reported above the same number of each cell was used for all injections ($1 \times 10^5$ of each). The tissues were fixed and stained for β-galactosidase activity first and then heated at 65°C for 30 min to reduce the background staining of lung tissue alkaline phosphatase activity (this was verified in control experiments). Finally, they were stained for alkaline phosphatase activity expressed from the transfected ALP gene in APSI cells.

As shown in Figure 3a–c at 1 h post-injection, the Red-gal and X-phosphate combination provided very good contrast between red-staining LZEJ foci (black arrowheads) and blue-staining APSI foci (black arrows). While most foci were homogeneously staining for the marker genes and well-separated from neighbouring foci, some double-staining foci were always noted (open arrows in Figure 3a and b), even at highest magnification (Figure 3c). Therefore, the different tumour cell classes do undergo some degree of co-localisation in the lung after intravenous co-injection (see below as well).

Quantitation of the three classes of foci (APSI-only; LZEJ-only; co-localised foci) reveals some interesting aspects of micrometastasis establishment with time. At 1 h post-injection, more than 500 double-stained foci (6–7% of all foci) were resolved out of a total number of 6–7,000 foci (Table I). The number of double-staining foci decreased to 122 at 6 h post-injection. By 24 h, 24 double-stained foci were observed. These are shown in Figure 4 – well-separated homogeneous foci of LZEJ or APSI cells in most cases (Figure 4a), foci of the two cell types neighbouring each other but not intermixing (Figure 4b), and intimately associated foci of both cell types (Figure 4c and d). When the percentages of APSI-only and APSI/LZEJ co-foci are compared to the values at the 1 h time point (Table I), it is clear that the co-localised foci are cleared at approximately the same rate as the APSI-only foci. This indicates that the co-localised foci do not necessarily have any stabilising advantage for the establishment of micrometastases in the lung.

To evaluate more rigorously whether the two tumour cell classes 'associate' with each other during micrometastasis, sections of lung were analysed at these various time points. Neutral red was used to counter-stain normal lung tissue for resolution of host organ cells without interfering with the two histochemical staining reactions for tumour cell identification. Because of counter-staining, the X-gal (LZEJ cells stain blue) and X-phosphate/NBT (APSI cells stain reddish-black) combination was more effective for resolution of the two tumour cell classes in sections. At 1 h post-injection lung sections reveal well-separated foci of each class (Figure 5a), as well as at other time points (Figure 5b,c and d). Furthermore, double-staining foci (open arrows) can be seen readily at 1 h post-injection (Figure 5b), at 6 h (Figure 5c), and at 24 h (Figure 5d). Furthermore, all foci identified of both cell

**Figure 4** Persistent co-localisation of LZEJ/APSI lung microfoci after 24 h. Lungs from mice given an i.v. injection of a mixture of $1 \times 10^5$ LZEJ cells and $1 \times 10^5$ APSI cells were recovered at 24 h post-injection, fixed and stained with Red-gal to detect *lacZ* gene activity, heat-treated, and finally stained with X-phosphate to detect ALP gene activity after heat-treating the tissue. **a**, This panel illustrates that well-isolated and homogeneous microfoci can be detected after labile foci of both cell types have been cleared from the lungs. Two red-staining LZEJ foci are indicated by black arrowheads and two individual blue-staining APSI foci by black arrows (Magnification, 100 ×). **b**, A red-staining LZEJ microfocus is seen adjacent to a blue-staining APSI microfocus (Magnification, 100 ×). **c** and **d** show other examples of double-staining microfoci that are readily identified at this time point (both magnifications, 100 ×).

**Figure 5** Lung sections revealing separated and co-localised microfoci soon after co-injection of LZEJ and APSI cells. At the indicated time points, lungs from mice injected i.v. with a mixture of $1 \times 10^5$ LZEJ and $1 \times 10^5$ APSI cells were stained with X-gal for *lacZ* activity and then with X-phosphate/NBT for ALP activity after heat treatment. Lungs were then embedded in methacrylate as described in Materials and methods and sections counter-stained with neutral red. Blue-staining LZEJ cells are indicated by black arrowheads and reddish-black staining APSI cells by black arrows. Double-stained foci containing both cell types are indicated by open arrows; the intimacy of the two tumour cell classes to each other is readily apparent in these sections. **a**, 1 h post-injection (Magnification, 360 ×). **b**, 1 h post-injection (Magnification, 360 ×). **c**, 6 h post-injection (Magnification, 360 ×). **d**, 24 h post-injection (360 ×).

classes contained several cells (usually 2–6 cells) and confirms earlier studies of LZEJ for the multi-cellularity of lung foci (Lin *et al.*, 1990*b*). Intimate tumour cell: tumour cell relationships are therefore revealed by histochemical analyses of lung sections.

Development of large metastatic nodules from micrometastases was then evaluated with co-injected populations. The quantitative data of Table I reveal that a number of the metastatic nodules during several weeks in the lung contain both APSI and LZEJ cells. Association of the two cell types in these expanding nodules is revealed by staining whole lungs at 3 weeks (Figure 6). Interspersed LZEJ (blue staining) and APSI (reddish-black staining) cells are apparent in some nodules (e.g. Figure 6a). In other cases, a large LZEJ nodule neighbours a small APSI micrometastasis (Figure 6b). APSI or LZEJ nodules can neighbour each other, but not be in intimate contact (Figure 6c) while in other cases may be contacting (Figure 6e). Another example of both cell types co-growing in a common metastatic nodule is provided in Figure 6f. Even at this later time, many foci persist as micrometastases (Figure 6d). These findings reveal that not all metastases are uniclonal – a significant number are at least biclonal (i.e. containing both LZEJ and APSI cells), raising the possibility that intercellular cooperation between the two cell types could facilitate experimental lung metastasis.

This paradigm was extended to two other experiments in which (a) the two cell classes are injected sequentially with a small period of time between the two injections or (b) one of the cell types in culture is replication-inactivated by Mitomycin C treatment prior to injection into the animal. Mitomycin C inhibits cell division while permitting them to stably express differentiated products as 'feeder layers' for several weeks in culture (Baroffio *et al.*, 1988; Lin & Culp, 1992*b*). Mitomycin C-treated cells were shown to be nonviable by their inability to form colonies in culture at low dilutions (data not shown).

Cooperation between LZEJ and APSI cells is indicated by the increased numbers of lung nodules in sequentially-injected animals (Table II; compare with APSI-only or APSI/LZEJ data at 3 weeks in Table I). When 6 h separate the two injections, the number of expanding nodules in the lung remains high for both cell types. This cannot be explained by a simple mass-action principle because injection of $2 \times 10^5$ cells of only APSI or LZEJ does not lead to a doubling of the number of metastatic nodules (data not shown). Moreover, when APSI cells are first rendered non-dividing with Mitomycin C (Lin & Culp, 1992*b*), the number of LZEJ nodules decreases from 19 to 11 and, as expected, APSI-only nodules become nonexistent. When the inter-injection time is increased to 7 days, there continues to be some complementation of the two cell types in forming LZEJ nodules; this is also abolished by treating cells with Mitomycin C prior to injection. Since Mitomycin C-treated APSI cells also implant in the lung for several hours similarly to untreated cells, these results cannot be explained by improved initiation of micrometastasis development for LZEJ cells by APSI cells. Rather, dividing APSI cells are required for facilitation. These data

**Figure 6** Co-development of lung metastases at later time points. At 3 weeks post-injection i.v., lungs were recovered from animals receiving a mixture of $1 \times 10^5$ LZEJ and $1 \times 10^5$ APSI cells (panels **a–e**) or of $1 \times 10^5$ LZEJ and $1 \times 10^5$ APB cells (panel **f**)). Lungs were fixed and stained with X-gal for *lacZ* activity and then with X-phosphate/NBT for ALP activity. Blue-staining LZEJ cells are indicated by black arrowheads; reddish-black staining APSI or APB cells by black arrows. **a**, A heterogeneous-staining small nodule contains APSI cells interspersed among more prominent LZEJ cells (Magnification, $100\times$). **b**, A small APSI focus indicated by a black arrow is observed adjacent to a large blue-staining LZEJ nodule (Magnification, $100\times$). **c**, A blue-staining LZEJ nodule is observed next to a reddish-black-staining APSI nodule; there does not appear to be any mixing of the two tumour cell classes in these separated nodules (Magnification, $100\times$). **d**, Two well-separated collections of micrometastatic foci are indicated (Magnification, $100\times$). **e**, A large APSI nodule, containing highly staining cells in some regions and non-staining cells in other regions, is abutting a smaller blue-staining LZEJ focus (Magnification, $100\times$). **f**, A heterogeneous-staining nodule contains both LZEJ and APB cells, while a nearby small nodule contains only APB cells (Magnification, $100\times$). Note that in the large nodule the APSI cells tend to concentrate at the periphery of the nodule while the LZEJ cells concentrate in the central region.

**Table II** Quantitation of metastasis formation by sequential injections of LZEJ/APSI cells[a]

| *Injection protocol* | *Time of sacrifice* | *Lung LZEJ-stained* | *Metastatic APSI-stained* | *Nodules Non-stained* |
|---|---|---|---|---|
| LZEJ - 6 h - APSI | 3 weeks | 19[c] **(1)**[d] | 19[c] **(1)**[d] | 6[c] |
| LZEJ - 6 h - APSI (Mitomycin C)[b] | 3 weeks | 11[c] | 0[c] | 3[c] |
| LZEJ - 7 days - APSI | 3 weeks | 19[c] | 0[c] | 3[c] |
| LZEJ - 7 days - APSI (Mitomycin C)[b] | 3 weeks | 8[c] | 0[c] | 6[c] |

[a]Mice (two for each datum value) were given i.v. injections of $1 \times 10^5$ LZEJ cells first, followed by the indicated time span with $1 \times 10^5$ APSI cells. Three weeks post-injection, mice were sacrificed. Whole lungs were removed, rinsed well with PBS, and stained with X-gal and X-phosphate/NBT for enumeration of the metastatic nodules detected in the lungs. In most cases, the nodules were homogeneously stained for only one marker gene, with the exception of double-staining nodules shown in parentheses. In a few cases, nodules contained segments that were stained for one of the marker genes and neighbouring segments that were unstained (**non-stained**). [b]APSI cells were treated with $5\ \mu g\ ml^{-1}$ Mitomycin C in culture (prior to their injection into animals) for 16 h in order to prevent any cell doubling while maintaining their ability to express the marker gene and other differentiation genes. [c]Number of foci (stained or unstained as indicated) per mouse per entire lung when all three lobes were analysed in this group. [d](#) = **double stained nodules**, containing both LZEJ and APSI cells co-localised in the same nodule.

suggest that stability/expandability of LZEJ pulmonary microfoci occurs many hours-to-days following their injection and that live APSI cells contribute environmental cues that improve these processes for LZEJ cells.

### Discussion

These analyses demonstrate the effectiveness of using two different histochemical marker genes to tag two different, but related (both being oncogene derivatives of clone A31 Balb/c 3T3 cells), tumour cell classes *in situ* and to evaluate whether the two classes alter the tumour progression characteristics of each other. They support the concept of multiple clonal interactions during tumour progression (Heppner, 1984; Hart & Saini, 1992). Evidence has been obtained for the phenotypic/genotypic heterogeneity of breast carcinoma tumour populations in animal model systems, as well as evidence for cooperativity among multiple subpopulations in the breast tumour system (Miller *et al.*, 1980, 1988). However, little study has addressed these possibilities in other tumour systems of human or animal origin. The approach described herein can be used for any two or three related tumour cell classes by transfecting different histochemical maker genes into each class to permit their precise identification in complex environments at the single-cell level. Each class could be generated by transfecting different oncogenes, tumour suppressor genes, or other candidate metastasis genes into a common parental cell to evaluate their significance during tumour progression.

Previous studies had demonstrated the utility of the bacterial *LacZ* gene for tracking EJ-Ha-*ras*-transformed Balb/c 3T3 cells during progression (Lin & Culp, 1992*a*; Lin *et al.*, 1990*a*,*b*). The current experiments illustrate the same utility and specificity of the human placental alkaline phosphatase gene (Lin & Culp, 1991), transfected into c-*sis* transformed 3T3 cells, for tracking APSI cells. With two different combinations (X-phosphate alone to stain cells blue or X-phosphate plus the cofactor NBT to stain them reddish-black), APSI cells stained intensely and selectively, with minimal staining of untagged 3T3, host organ cells, or LZEJ cells *in vitro* or *in situ*. Furthermore, double-staining protocols were effective for discriminating mixed populations of the two cell classes in culture, in whole organ preparations, or in organ sections.

APSI cells were less tumorigenic than LZEJ cells, displaying a longer latency of primary tumour development after subcutaneous injection and an inability to metastasise to the lung. The fate of APSI cells was also followed during the first 24 h after intravenous injection, since this is the critical period for establishment and/or clearance of many tumour cells during experimental metastasis (Fidler & Balch, 1987; Nicholson, 1988), as also shown for tumour cell LZEJ (Lin *et al.*, 1990*b*). The kinetics of APSI clearance differed somewhat from those of LZEJ – the vast majority of APSI cells were cleared within the first 6 h while a sizeable fraction of LZEJ cells were cleared after this, but prior to the basal level observed at 24 h. APSI micrometastases that became established by 24 h appeared to localise at the ends of the smallest blood vessels in the lung and were always comprised of 2–6 cells. Therefore, multi-cellularity of lung foci occurs for 3T3 cells transfected with two different oncogenes. Blood vessels in lung can be detected by a longer period of fixation prior to histochemical staining, facilitating retention of hemoglobin-containing cells. Some APSI micrometastases expanded into nodules, some of which stained homogeneously for ALP gene activity and some of which were heterogeneous in their staining because of $ALP^-$ variants. The basis for loss of marker gene expression in variants remains for exploration; this property may prove useful for quantitating the complex evolution of clonal variants during tumour progression (Lin & Culp, 1992*a*). The complexity of clonal evolution of tumours is indicated with studies using random integration into human tumour cell DNA of drug resistance genes in experimental animal systems (Moffett *et al.*, 1992).

When LZEJ and APSI cells were co-injected intravenously, several pieces of evidence suggest that there may be facilitation of experimental metastasis by these two cell types. First, a significant fraction of the two cell types co-localise into foci at all time points providing opportunity for them to interact intimately in these tissue sites. This was confirmed by staining sections of lung for the two marker genes. Second, these foci persist in relative proportion to APSI-only or LZEJ-only foci from 1 h to several weeks post-injection, indicating that these co-localised foci are not selected for/against during the clearance/establishment events. Since APSI foci are cleared from the lung more efficiently than LZEJ foci after single injections, these results suggest that LZEJ cells may assist the survivability of APSI cells in the lung as a result of co-localisation. Third, the number of APSI-containing metastatic nodules increases when the two cell types are co-injected, when compared to APSI cells injected alone. This argues that LZEJ cells promote the metastatic competence of APSI cells, a concept which is also supported by evidence for both cell types being in a significant number of larger nodules (5–10%). Therefore, a significant fraction of lung metastases have a biclonal origin as a result of coordinate growth of both cell types in the same nodules; whether this provides further metastatic aggression and poorer survival for the animal remains to be determined. Fourth, sequential injection experiments also led to a doubling of nodules at 3 weeks containing APSI cells, when compared to the number of nodules when APSI cells were injected alone. Finally, when Mitomycin C-treated APSI cells were co-injected with live LZEJ cells, the number of lung nodules at 3 weeks post-injection containing LZEJ cells decreased by half. This result indicates that APSI cells can also promote metastatic competence of LZEJ cells, but only as a population of dividing cells. All of these data support a model of intercellular and reciprocal cooperation between the two tumour cell classes, both of which are derived from the same parent cell. They also support the hypothesis that two (or more) cell variants can be generated during 'natural' tumour expansion and that these variants might interact to promote tumour progression.

Although it is possible that PDGF produced by APSI cells may assist cell division of LZEJ cells, other molecular mechanisms must be tested as well. This is particularly relevant from indications that LZEJ cells facilitate progression of APSI cells. In this regard, there are several documented cases where growth factors – e.g. PDGF – promote tumour progression by a paracrine mechanism (Breillout *et al.*, 1989; Bronzert *et al.*, 1987; Egan *et al.*, 1990; Frazier *et al.*, 1988; Tsuruo *et al.*, 1989). *Sis*-oncogene-transformed cells secrete PDGF that stimulates division of neighbouring cells in a paracrine fashion (Johnson *et al.*, 1985), in addition to autocrine growth stimulation of transformed cells themselves (Keating & Williams, 1988). PDGF may have regulatory influence in other steps of tumour progression – during wound healing processes subsequent to invasion by micrometastatic cells; during activation of macrophages/monocytes during invasion/repair; and during vasoconstriction mediated by PDGF. Whether LZEJ cells secrete some soluble growth factor to promote expansion of APSI-containing micrometastases or whether they generate extracellular matrix products or some other product for this facilitation remains for future investigation.

Cooperation between *cis*-acting oncogenes (i.e. acting within the same cell) has been demonstrated in many systems. A nuclear-acting oncogene (e.g. *myc*) complements the activity of a cytoplasmic-acting oncogene (e.g. *ras*) to generate full metastatic competence (Weinberg, 1985). In some tumour progression studies, certain combinations of oncogenes suppress the metastatic phenotype (e.g. adenovirus Ela and *ras*) (Pozzatti *et al.*, 1988). The use of alternative histochemical marker genes now permits more careful evaluation of the significance for tumour progression of two related cell types whose gene products may act in *trans*. They will also permit testing of possible cooperation and/or inhibition between two cell types containing other classes of genes suspected of being critical for tumour progression.

The authors extend appreciation to Drs Theresa and Thomas Pretlow of the Department of Pathology, as well as Mary Ann O'Riordan of their laboratory, for assistance with histological analyses of sections and staining protocols.

### References

ASLAKSON, C.J., RAK, J.W., MILLER, B.E. & MILLER, F.R. (1991). Differential influence of organ site on three subpopulations of a single mouse mammary tumor at two distinct steps in metastasis. *Int. J. Cancer*, **47**, 466–472.

BARBACID, M. (1987). *ras* genes. *Annu. Rev. Biochem.*, **56**, 779–827.

BAROFFIO, A., DUPIN, E. & LEDUARIN, N.M. (1988). Clone-forming ability and differentiation potential of migratory neural crest cells. *Proc. Natl Acad. Sci. USA*, **85**, 5325–5329.

BECKMANN, M.P., BETSHOLTA, C., HELDIN, C.-H., WESTERMARK, B., MARCO, E.D., DI FIORE, P.P., ROBBINS, K.C. & AARONSON, S.A. (1988). Comparison of biological properties and transforming potential of human PDGF-A and PDGF-B chains. *Science*, **241**, 1346–1349.

BREILLOUT, F., ANTOINE, E., LASCAUZ, V., ROLLAND, Y. & POUPON, M.-F. (1989). Promotion of micrometastasis formation in a rat rhabodomyosarcoma model by epidermal growth factor. *J. Natl Cancer Inst.*, **81**, 702–705.

BRONZERT, D.A., PANTAZIS, P., ANTONIADES, H.N., KASID, A., DAVIDSON, N., DICKSON, R.B. & LIPPMAN, M.E. (1987). Synthesis and secretion of platelet-derived growth factor by human breast cancer cell lines. *Proc. Natl Acad. Sci. USA*, **84**, 5763–5767.

CAVANAUGH, P.G. & NICOLSON, G.L. (1989). Purification and some properties of a lung-derived growth factor that differentially stimulates the growth of tumor cells metastatic to the lung. *Cancer Res.*, **49**, 3928–3933.

EGAN, S.E., JAROLIM, L., ROGELJ, S., SPEARMAN, M., WRIGHT, J.A. & GREENBERG, A.H. (1990). Growth factor modulation of metastatic lung colonization. *Anticancer Res.*, **10**, 1341–1346.

EGAN, S.E., MCCLARTY, G.A., JAROLIM, L., WRIGHT, J.A., SPIRO, I., HAGER, G. & GREENBERG, A.H. (1987). Expression of H-*ras* correlates with metastatic potential: evidence for direct regulation of the metastatic phenotype in 10T1/2 and NIH 3T3 cells. *Mol. Cell. Biol.*, **7**, 830–837.

FIDLER, I.J. & BALCH, C.M. (1987). The biology of cancer metastasis and implications for therapy. *Curr. Probl. Surg.*, **24**, 129–209.

FRAZIER, G.C., BOWEN-POPE, D.F., SEIFERT, R.A. & VOGEL, A.M. (1988). Association of increased platelet-derived growth factor secretion and retroviral expression in transformed mouse and human cells. *Cancer Res.*, **48**, 4874–4880.

GOUSTIN, A.S., LEOF, E.B., SHIPLEY, G.D. & MOSES, H.L. (1986). Growth factors and cancer. *Cancer Res.*, **46**, 1015–1029.

HART, I.R., GOODE, N.T. & WILSON, R.E. (1989). Molecular aspects of the metastatic cascade. *Biochim. Biophys. Acta*, **989**, 65–84.

HART, I.R. & SAINI, A. (1992). Biology of tumor metastasis. *Lancet*, **339**, 1453–1457.

HENTHORN, P., ZERVOS, P., RADUCHA, M., HARRIS, H. & KADESCH, T. (1988). Expression of a human placental alkaline phosphatase gene in transfected cells: use as a reporter for studies of gene expression. *Proc. Natl Acad. Sci. USA*, **85**, 6342–6346.

HEPPNER, G.H. (1984). Tumor heterogeneity. *Cancer Res.*, **44**, 2259–2265.

HEPPNER, G.H. & MILLER, B.E. (1983). Tumor heterogeneity: biological implications and therapeutic consequences. *Cancer Metastasis Rev.*, **2**, 5–23.

JOHNSON, A., BETSHOLTZ, C., VON DER HEM, K., HELDIN, C.-H. & WESTERMARK, B. (1985). Platelet-derived growth factor agonist activity of a secreted form of the v-*sis* oncogene product. *Proc. Natl Acad. Sci. USA*, **82**, 1721–1725.

KEATING, M.T. & WILLIAMS, L.T. (1988). Autocrine stimulation of intracellular PDGF receptors in v-*sis* transformed cells. *Science*, **239**, 914–916.

LIN, W.-C. & CULP, L.A. (1991). Selectable plasmid vectors with alternative and ultrasensitive histochemical marker genes. *BioTechniques*, **11**, 344–351.

LIN, W.-C. & CULP, L.A. (1992*a*). New insights into micrometastasis development using ultrasensitive marker genes. In *Current Perspectives on Molecular and Cellular Oncology*, Spandidos, D. (ed.), Vol. 1, Part B, pp. 261–309. London: JAI Press, Ltd.

LIN, W.-C. & CULP, L.A. (1992*b*). Altered establishment/clearance mechanisms during experimental micrometastasis with live and/or disabled bacterial *lacZ*-tagged tumor cells. *Invasion & Metastasis* **12**, 197–209.

LIN, W.-C., PRETLOW, T.P., PRETLOW, T.G. II & CULP, L.A. (1990*a*). Bacterial *lacZ* gene as a highly sensitive marker to detect micrometastasis formation during tumor progression. *Cancer Res.*, **50**, 2808–2817.

LIN, W.-C., PRETLOW, T.P., PRETLOW, T.G. II & CULP, L.A. (1990*b*). Development of micrometastasis: earliest events analyzed with bacterial *lacZ*-tagged tumor cells. *J. Natl Cancer Inst.*, **82**, 1497–1503.

LIN, W.-C., PRETLOW, T.P., PRETLOW, T.G. II & CULP, L.A. (1992) High resolution analyses of two different classes of tumor cells *in situ* tagged with alternative histochemical marker genes. *Am. J. Pathol.*, **141**, 1331–1342.

MAGHAZACHI, A.A., HERBERMAN, R.B., VUJANOVIC, N.L. & HISERODT, J.C. (1988). *In vivo* distribution and tissue localization of highly purified rat lymphokine-activated killer (LAK) cells. *Cellular Immunology*, **115**, 179–194.

MILLER, B.E., MILLER, F.R., LEITH, J. & HEPPNER, G.H. (1980). Growth interaction *in vivo* between tumor subpopulations derived from a single mouse mammary tumor. *Cancer Res.*, **40**, 3977–3981.

MILLER, B.E., MILLER, F.R., WILBURN, D. & HEPPNER, G.H. (1988). Dominance of a tumor subpopulation line in mixed heterogeneous mouse mammary tumors. *Cancer Res.*, **48**, 5747–5753.

MILLER, F.R. & HEPPNER, G.H. (1990). Cellular interactions in metastasis. *Cancer Metastasis Rev.*, **9**, 21–34.

MOFFETT, B.F., BABAN, D., BAO, L. & TARIN, D. (1992). Fate of clonal lineages during neoplasia and metastasis studied with an incorporated genetic marker. *Cancer Res.*, **52**, 1737–1743.

NICOLSON, G.L. (1988). Cancer metastasis: tumor cell and host organ properties important in metastasis to specific secondary sites. *Biochim. Biophys. Acta*, **948**, 175–224.

POZZATTI, R., MCCORMICK, M., THOMPSON, M.A. & KHOURY, G. (1988). The Ela gene of adenovirus type 2 reduces the metastatic potential of *ras*-transformed rat embryo cells. *Molecular and Cellular Biology*, **8**, 2984–2988.

RADINSKY, R. & CULP, L.A. (1991). Clonal dominance of select subsets of viral Kirsten $ras^{+}$-transformed 3T3 cells during tumor progression. *Int. J. Cancer*, **48**, 148–159.

RADINSKY, R., KRAEMER, P.M., RAINES, M.A., KUNG, H.-J. & CULP, L.A. (1987). Amplification and rearrangement of the Kristen *ras* oncogene in virus-transformed Balb/c 3T3 cells during malignant tumor progression. *Proc. Natl Acad. Sci. USA*, **84**, 5143–5147.

SUGITA, K., KOIZUMI, K. & YOSHIDA, H. (1992). Morphological reversion of *sis*-transformed NIH 3T3 cells by trichostatin A. *Cancer Res.*, **52**, 168–172.

TSURUO, T., WATANABE, M. & O'HARA, T. (1989). Stimulation of the growth of metastatic clones of mouse colon adenocarcinoma 26 by platelet-derived growth factor. *Jpn. J. Cancer Res.*, **80**, 136–140.

WEINBERG, R. (1985). The action of oncogenes in the cytoplasm and nucleus. *Science*, **230**, 770–776.

WESTERMARK, B. & HELDIN, C.-H. (1991). Platelet-derived growth factor in autocrine transformation. *Cancer Res.*, **64**, 271–280.

ZHAN, X. & GOLDFARB, M. (1986). Growth factor requirements of oncogene-transformed NIH3T3 and Balb/c 3T3 cells in defined media. *Mol. Cell. Biol.*, **6**, 3541–3544.


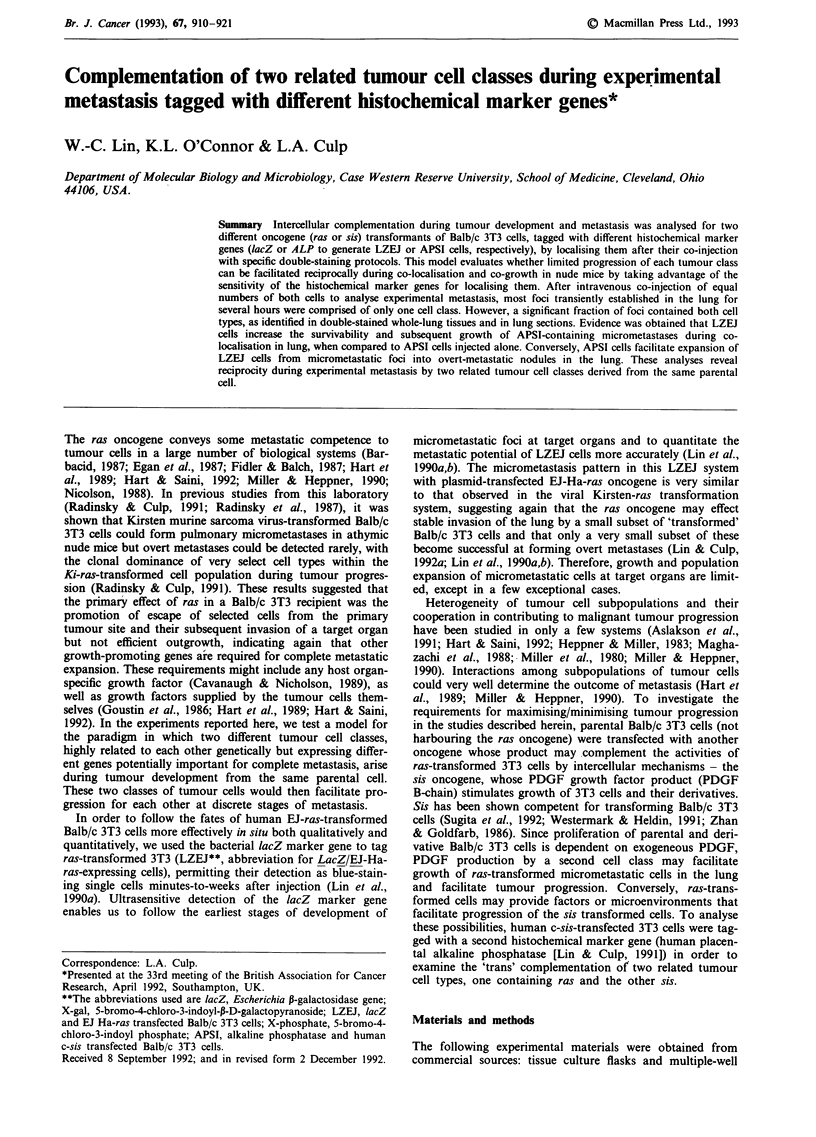

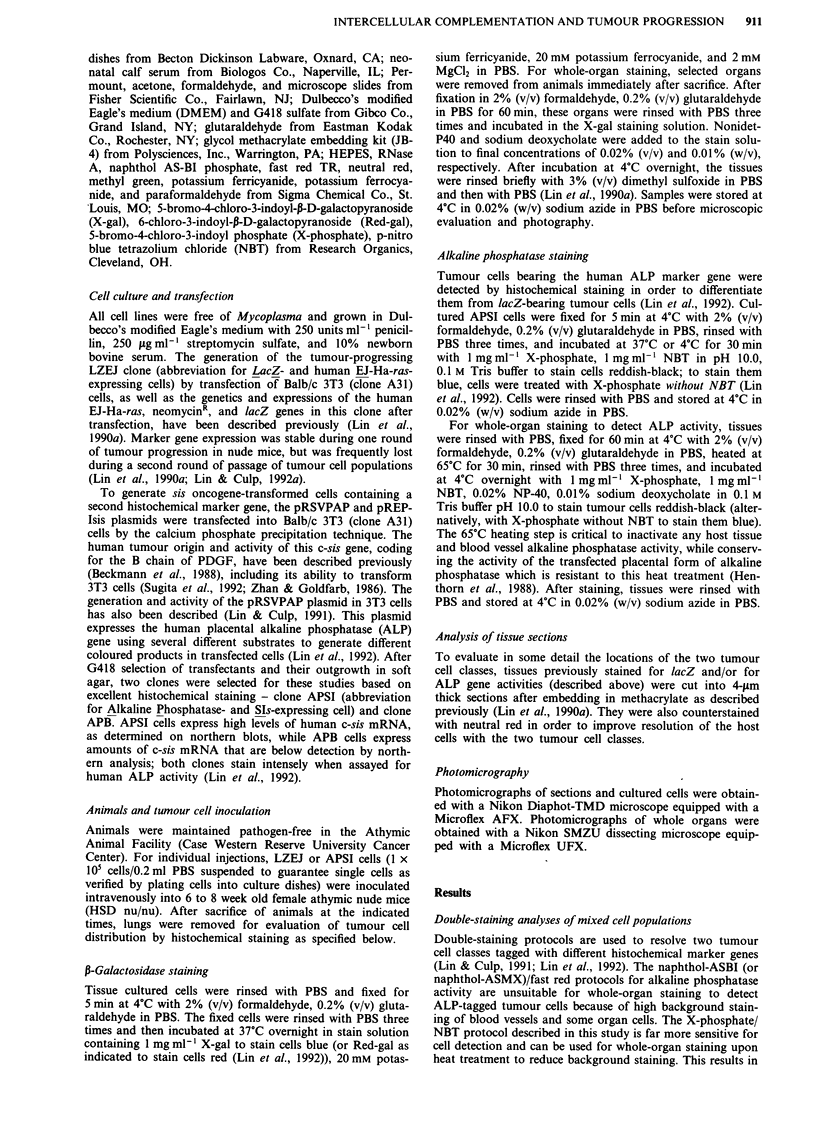

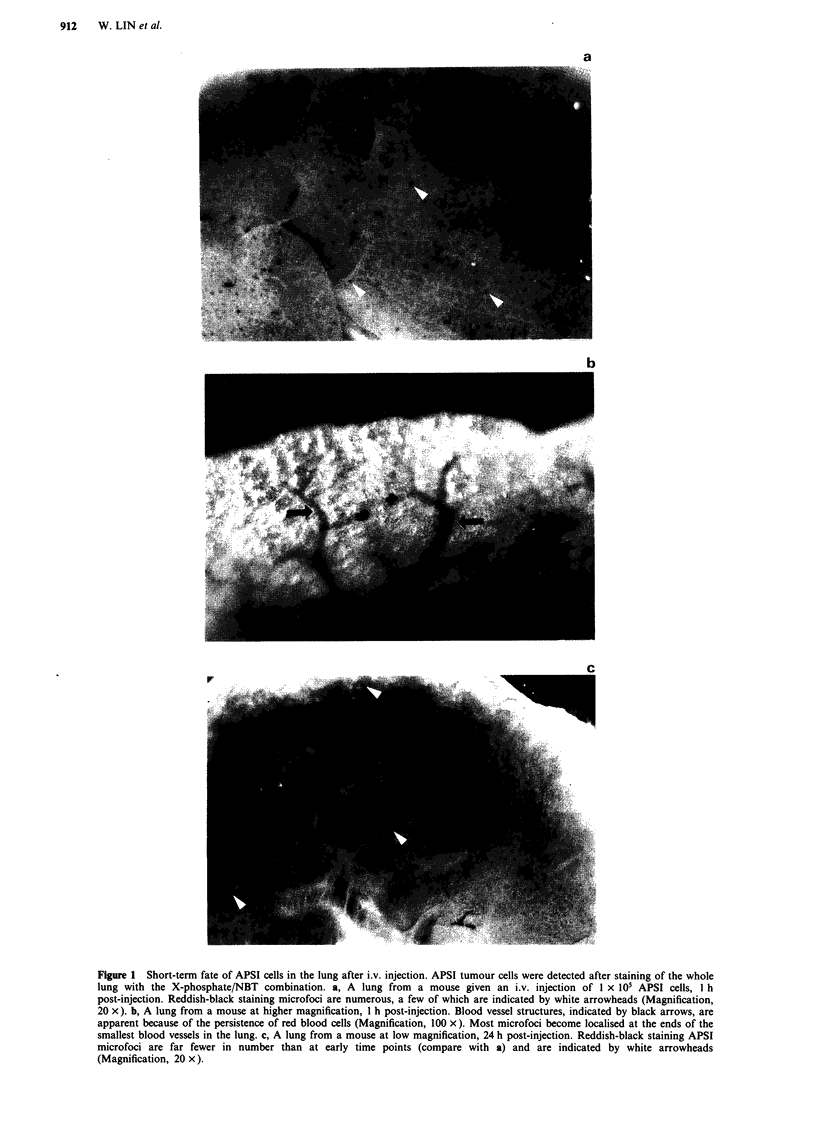

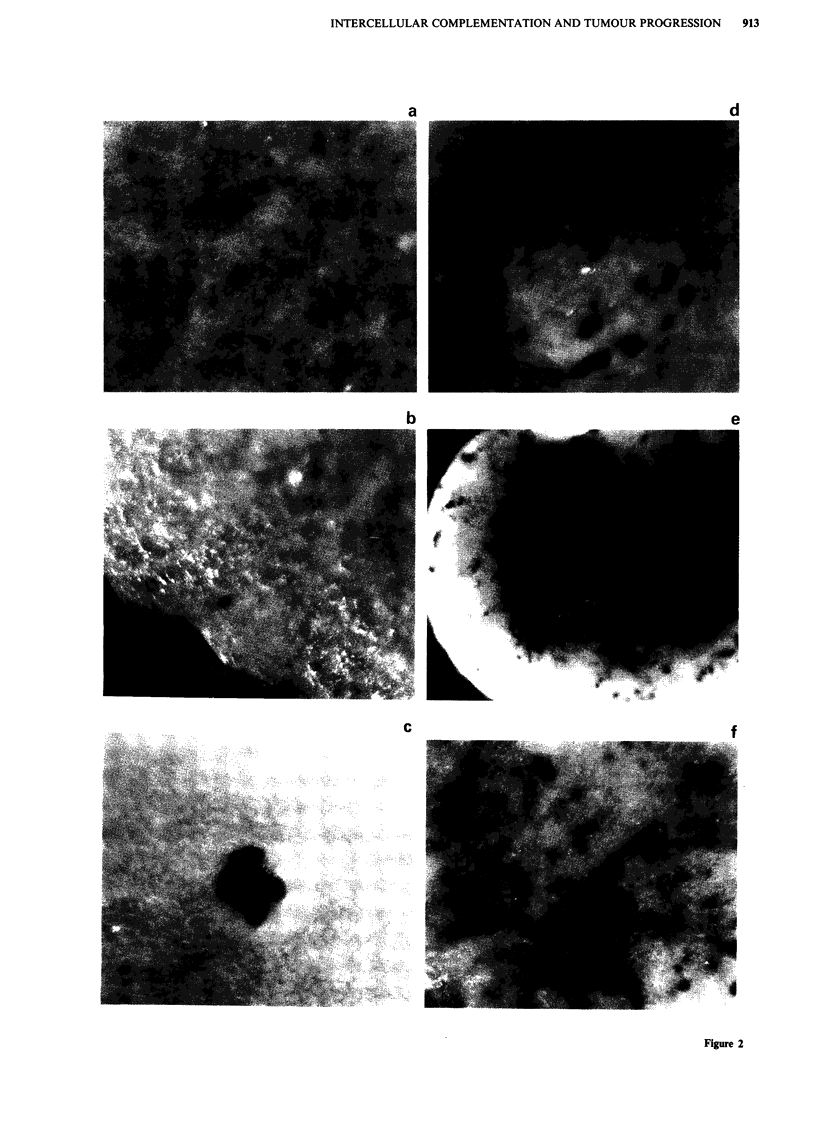

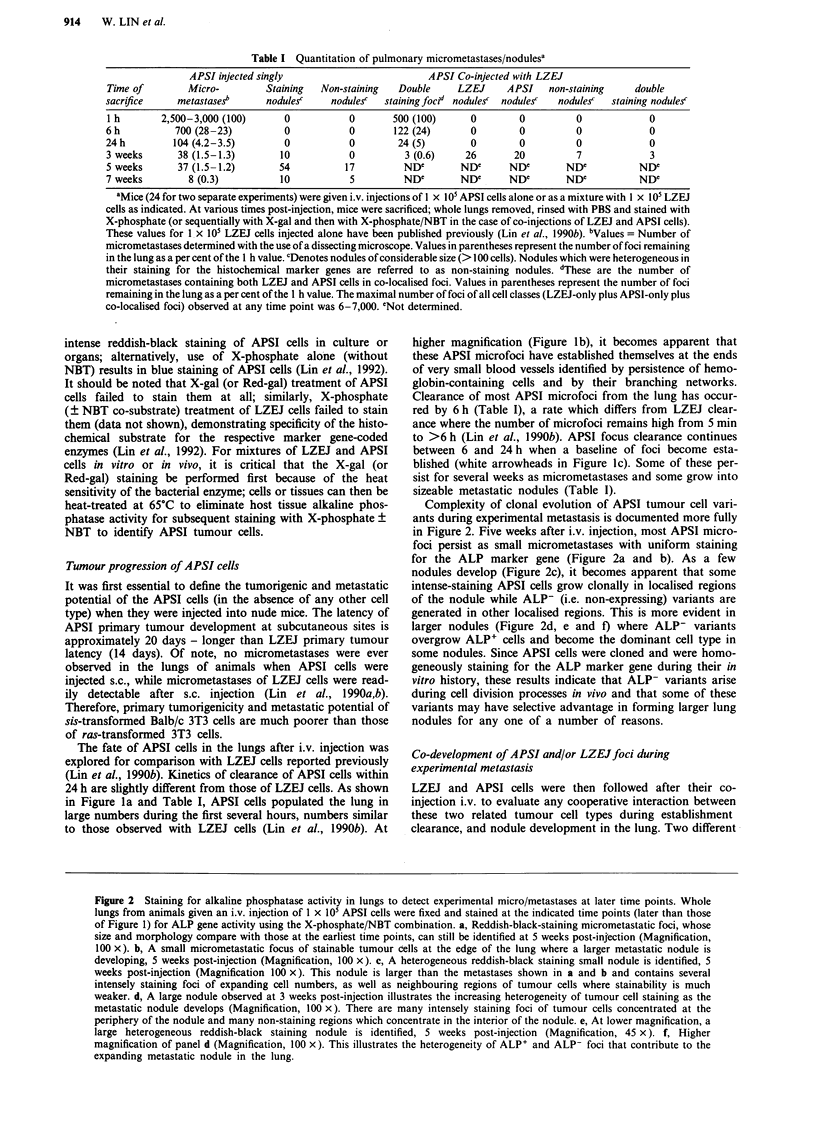

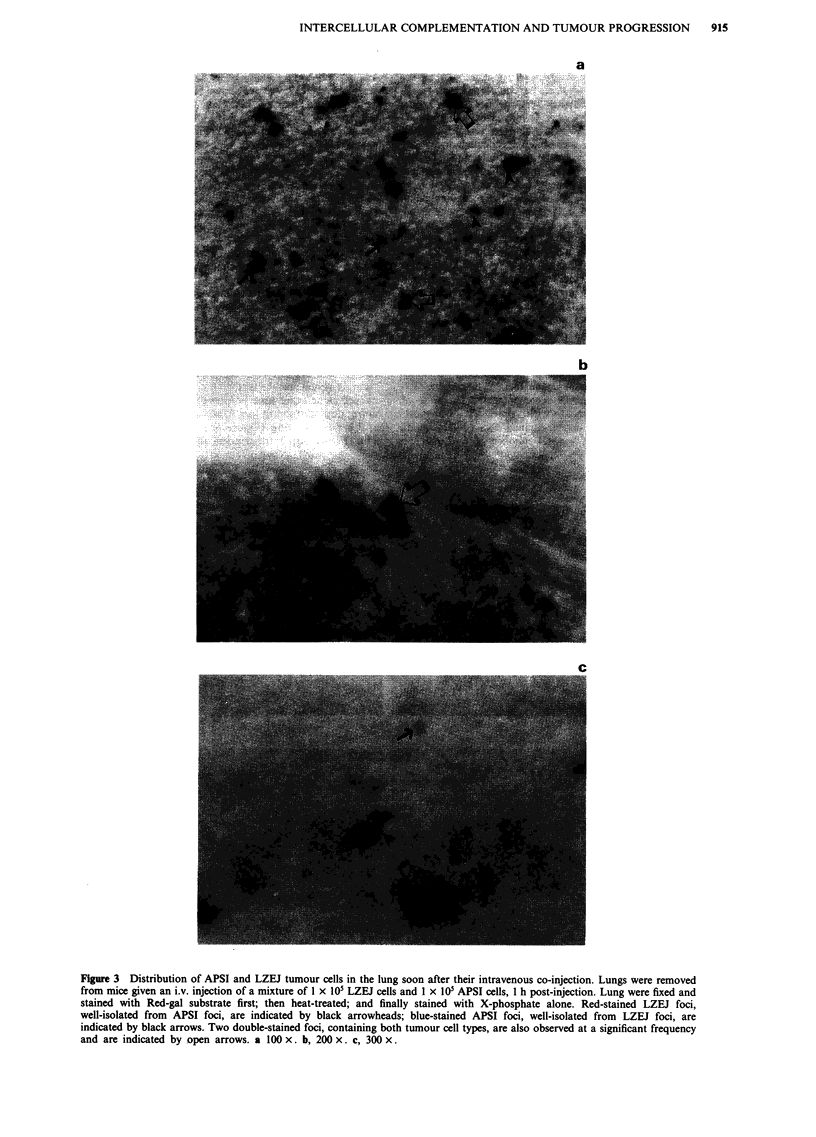

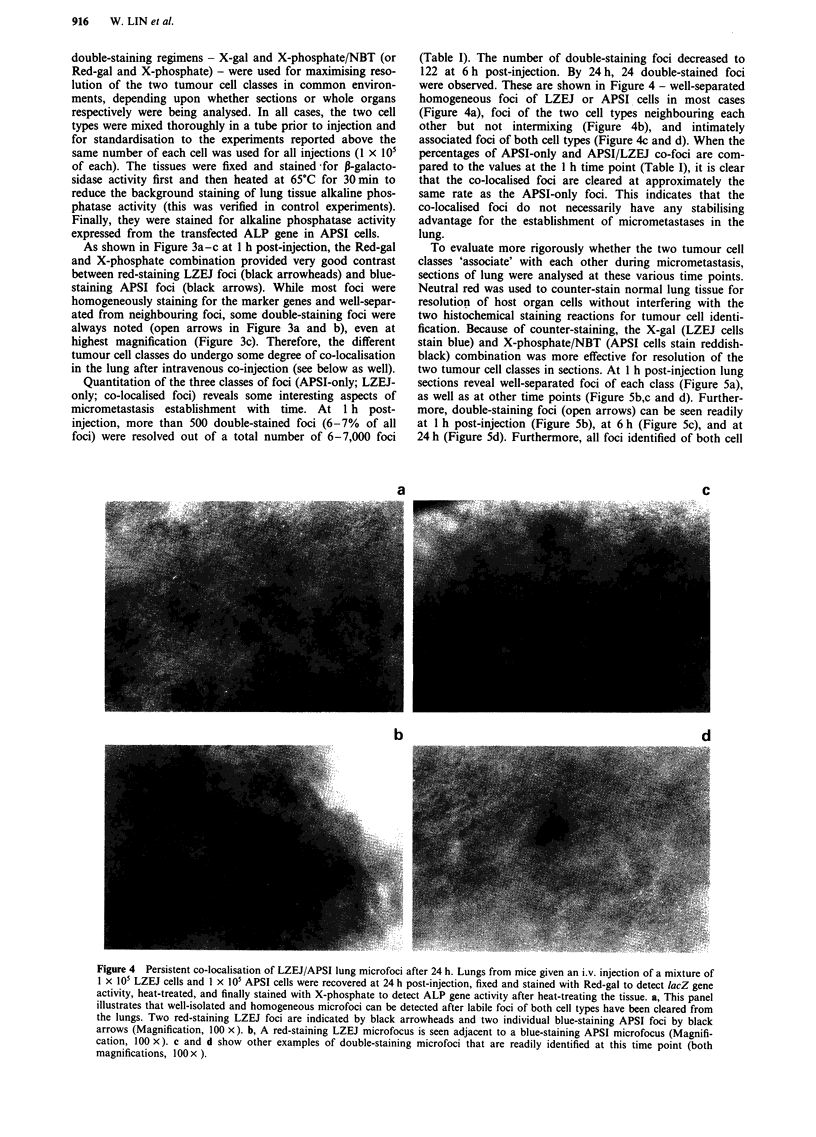

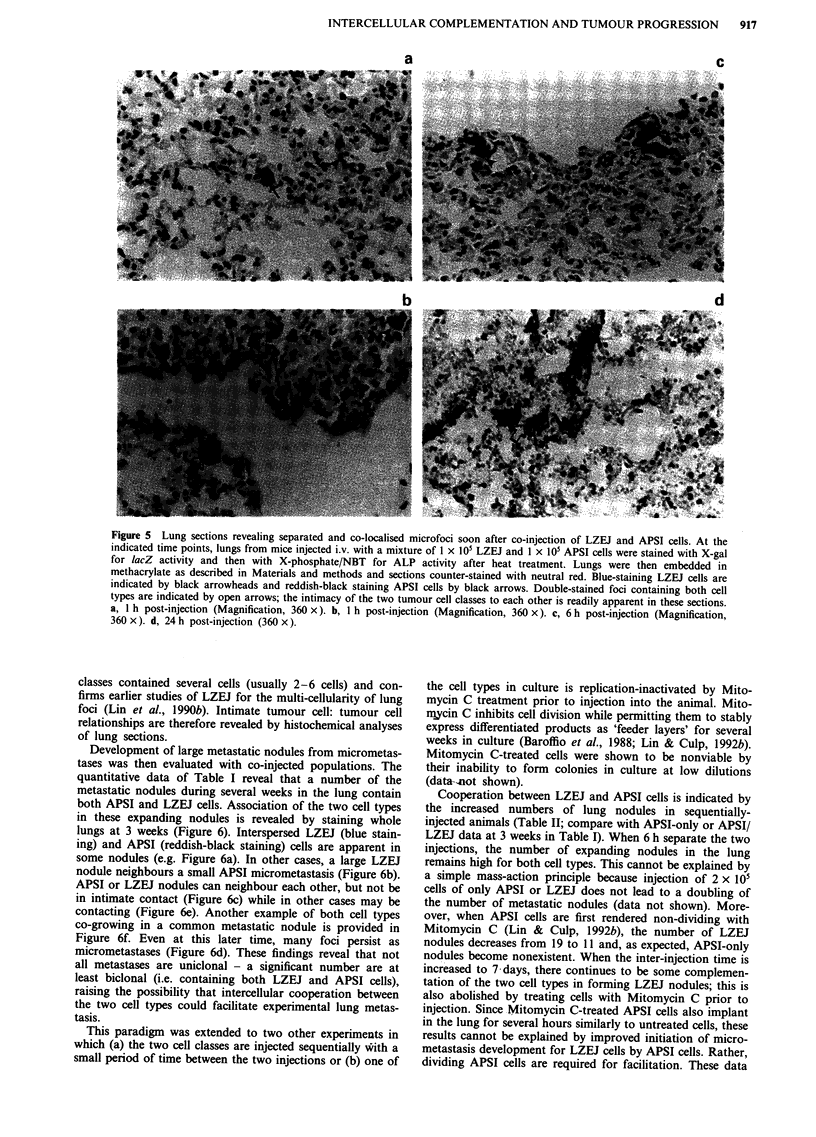

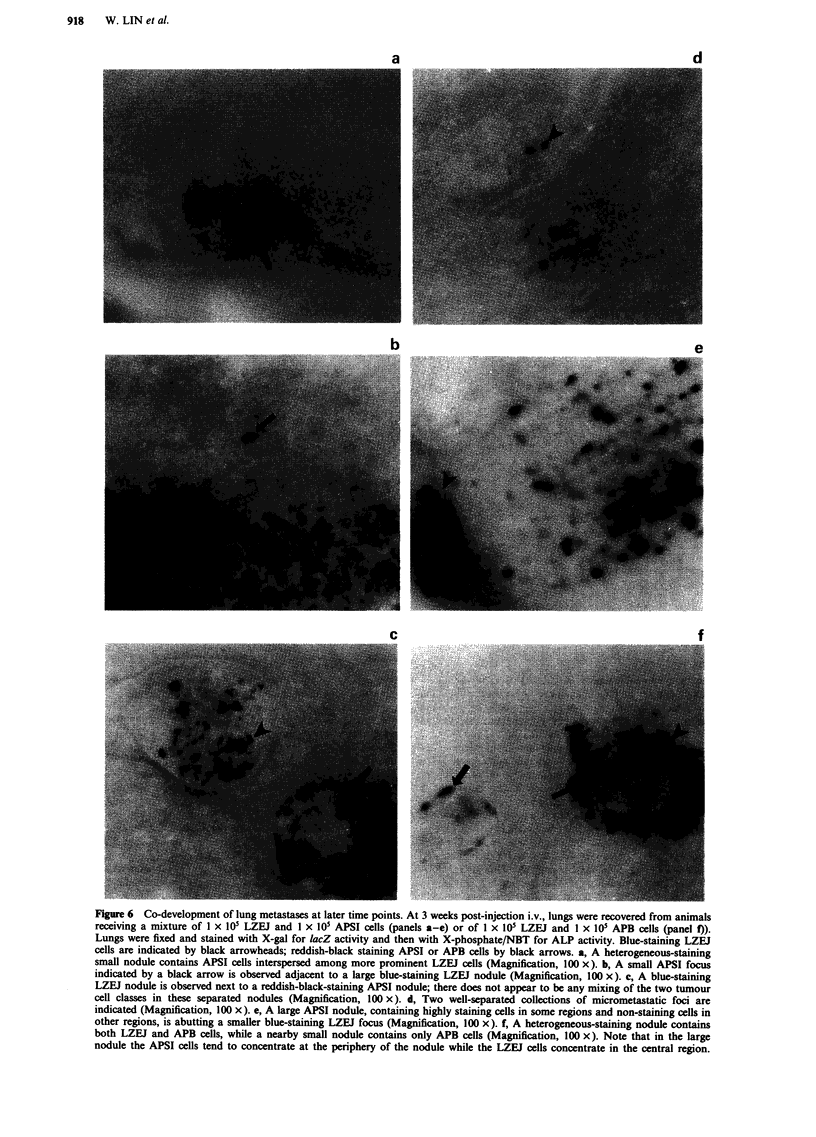

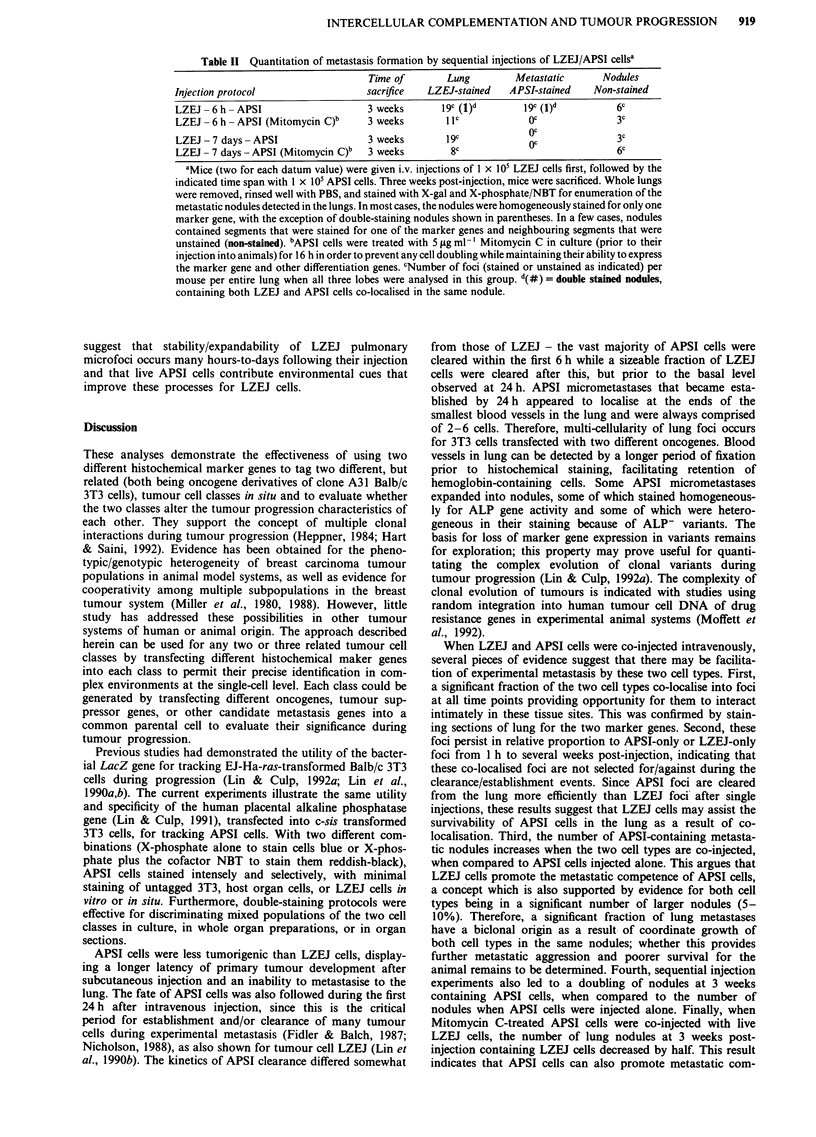

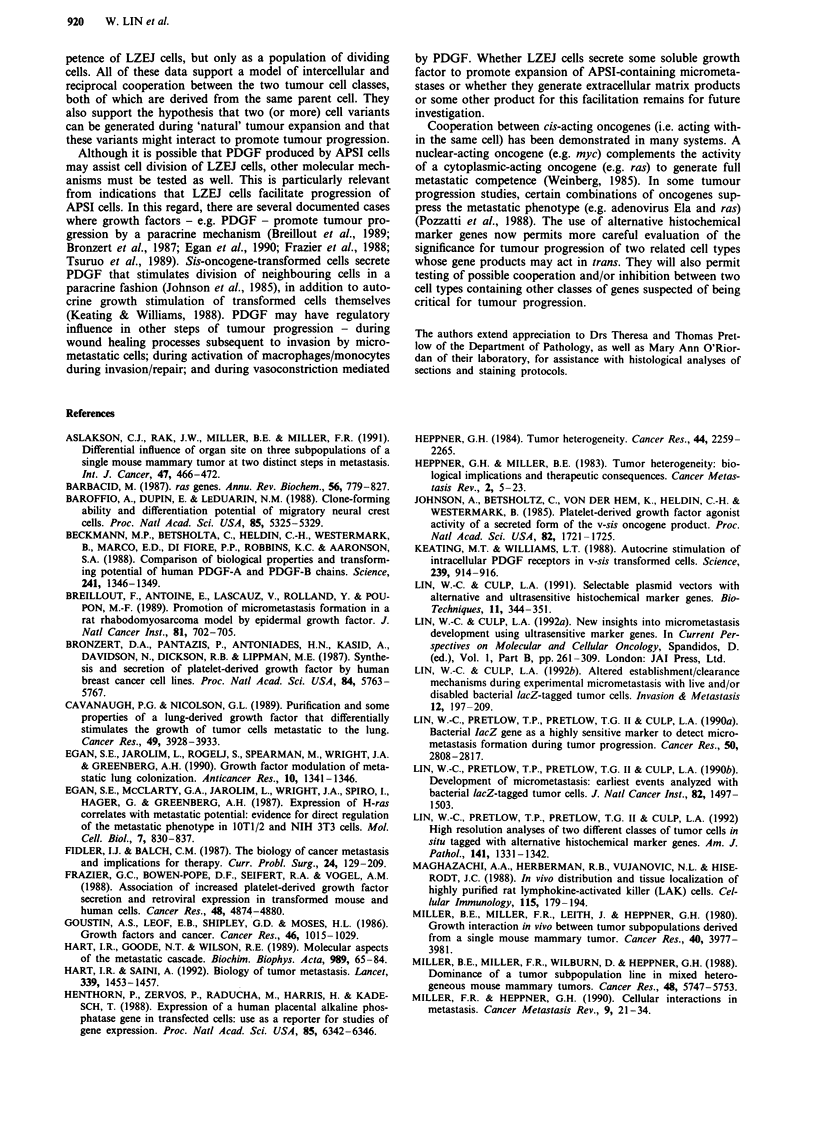

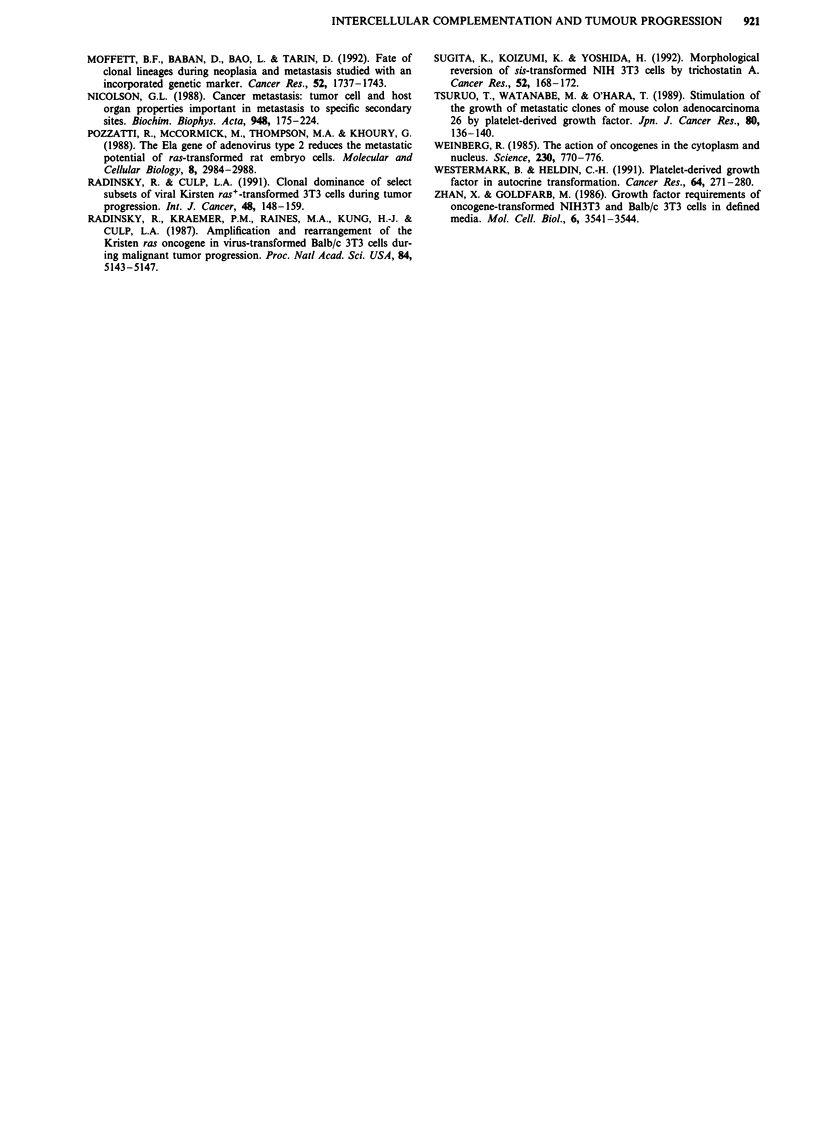


## References

[OCR_01010] Aslakson C. J., Rak J. W., Miller B. E., Miller F. R. (1991). Differential influence of organ site on three subpopulations of a single mouse mammary tumor at two distinct steps in metastasis.. Int J Cancer.

[OCR_01016] Barbacid M. (1987). ras genes.. Annu Rev Biochem.

[OCR_01017] Baroffio A., Dupin E., Le Douarin N. M. (1988). Clone-forming ability and differentiation potential of migratory neural crest cells.. Proc Natl Acad Sci U S A.

[OCR_01022] Beckmann M. P., Betsholtz C., Heldin C. H., Westermark B., Di Marco E., Di Fiore P. P., Robbins K. C., Aaronson S. A. (1988). Comparison of biological properties and transforming potential of human PDGF-A and PDGF-B chains.. Science.

[OCR_01031] Breillout F., Antoine E., Lascaux V., Rolland Y., Poupon M. F. (1989). Promotion of micrometastasis proliferation in a rat rhabdomyosarcoma model by epidermal growth factor.. J Natl Cancer Inst.

[OCR_01035] Bronzert D. A., Pantazis P., Antoniades H. N., Kasid A., Davidson N., Dickson R. B., Lippman M. E. (1987). Synthesis and secretion of platelet-derived growth factor by human breast cancer cell lines.. Proc Natl Acad Sci U S A.

[OCR_01042] Cavanaugh P. G., Nicolson G. L. (1989). Purification and some properties of a lung-derived growth factor that differentially stimulates the growth of tumor cells metastatic to the lung.. Cancer Res.

[OCR_01048] Egan S. E., Jarolim L., Rogelj S., Spearman M., Wright J. A., Greenberg A. H. (1990). Growth factor modulation of metastatic lung colonization.. Anticancer Res.

[OCR_01053] Egan S. E., McClarty G. A., Jarolim L., Wright J. A., Spiro I., Hager G., Greenberg A. H. (1987). Expression of H-ras correlates with metastatic potential: evidence for direct regulation of the metastatic phenotype in 10T1/2 and NIH 3T3 cells.. Mol Cell Biol.

[OCR_01060] Fidler I. J., Balch C. M. (1987). The biology of cancer metastasis and implications for therapy.. Curr Probl Surg.

[OCR_01063] Fraizer G. C., Bowen-Pope D. F., Seifert R. A., Vogel A. M. (1988). Association of increased platelet-derived growth factor secretion and retroviral expression in transformed mouse and human cells.. Cancer Res.

[OCR_01069] Goustin A. S., Leof E. B., Shipley G. D., Moses H. L. (1986). Growth factors and cancer.. Cancer Res.

[OCR_01073] Hart I. R., Goode N. T., Wilson R. E. (1989). Molecular aspects of the metastatic cascade.. Biochim Biophys Acta.

[OCR_01076] Hart I. R., Saini A. (1992). Biology of tumour metastasis.. Lancet.

[OCR_01082] Henthorn P., Zervos P., Raducha M., Harris H., Kadesch T. (1988). Expression of a human placental alkaline phosphatase gene in transfected cells: use as a reporter for studies of gene expression.. Proc Natl Acad Sci U S A.

[OCR_01090] Heppner G. H., Miller B. E. (1983). Tumor heterogeneity: biological implications and therapeutic consequences.. Cancer Metastasis Rev.

[OCR_01086] Heppner G. H. (1984). Tumor heterogeneity.. Cancer Res.

[OCR_01095] Johnsson A., Betsholtz C., von der Helm K., Heldin C. H., Westermark B. (1985). Platelet-derived growth factor agonist activity of a secreted form of the v-sis oncogene product.. Proc Natl Acad Sci U S A.

[OCR_01101] Keating M. T., Williams L. T. (1988). Autocrine stimulation of intracellular PDGF receptors in v-sis-transformed cells.. Science.

[OCR_01117] Lin W. C., Culp L. A. (1992). Altered establishment/clearance mechanisms during experimental micrometastasis with live and/or disabled bacterial lacZ-tagged tumor cells.. Invasion Metastasis.

[OCR_01106] Lin W. C., Culp L. A. (1991). Selectable plasmid vectors with alternative and ultrasensitive histochemical marker genes.. Biotechniques.

[OCR_01123] Lin W. C., Pretlow T. P., Pretlow T. G., Culp L. A. (1990). Bacterial lacZ gene as a highly sensitive marker to detect micrometastasis formation during tumor progression.. Cancer Res.

[OCR_01135] Lin W. C., Pretlow T. P., Pretlow T. G., Culp L. A. (1992). High-resolution analyses of two different classes of tumor cells in situ tagged with alternative histochemical marker genes.. Am J Pathol.

[OCR_01129] Lin W. C., Pretlow T. P., Pretlow T. G., Culp L. A. (1990). Development of micrometastases: earliest events detected with bacterial lacZ gene-tagged tumor cells.. J Natl Cancer Inst.

[OCR_01143] Maghazachi A. A., Herberman R. B., Vujanovic N. L., Hiserodt J. C. (1988). In vivo distribution and tissue localization of highly purified rat lymphokine-activated killer (LAK) cells.. Cell Immunol.

[OCR_01147] Miller B. E., Miller F. R., Leith J., Heppner G. H. (1980). Growth interaction in vivo between tumor subpopulations derived from a single mouse mammary tumor.. Cancer Res.

[OCR_01153] Miller B. E., Miller F. R., Wilburn D., Heppner G. H. (1988). Dominance of a tumor subpopulation line in mixed heterogeneous mouse mammary tumors.. Cancer Res.

[OCR_01157] Miller F. R., Heppner G. H. (1990). Cellular interactions in metastasis.. Cancer Metastasis Rev.

[OCR_01163] Moffett B. F., Baban D., Bao L., Tarin D. (1992). Fate of clonal lineages during neoplasia and metastasis studied with an incorporated genetic marker.. Cancer Res.

[OCR_01168] Nicolson G. L. (1988). Cancer metastasis: tumor cell and host organ properties important in metastasis to specific secondary sites.. Biochim Biophys Acta.

[OCR_01173] Pozzatti R., McCormick M., Thompson M. A., Khoury G. (1988). The E1a gene of adenovirus type 2 reduces the metastatic potential of ras-transformed rat embryo cells.. Mol Cell Biol.

[OCR_01179] Radinsky R., Culp L. A. (1991). Clonal dominance of select subsets of viral Kirsten ras(+)-transformed 3T3 cells during tumor progression.. Int J Cancer.

[OCR_01184] Radinsky R., Kraemer P. M., Raines M. A., Kung H. J., Culp L. A. (1987). Amplification and rearrangement of the Kirsten ras oncogene in virus-transformed BALB/c 3T3 cells during malignant tumor progression.. Proc Natl Acad Sci U S A.

[OCR_01191] Sugita K., Koizumi K., Yoshida H. (1992). Morphological reversion of sis-transformed NIH3T3 cells by trichostatin A.. Cancer Res.

[OCR_01196] Tsuruo T., Watanabe M., Oh-hara T. (1989). Stimulation of the growth of metastatic clones of mouse colon adenocarcinoma 26 in vitro by platelet-derived growth factor.. Jpn J Cancer Res.

[OCR_01202] Weinberg R. A. (1985). The action of oncogenes in the cytoplasm and nucleus.. Science.

[OCR_01210] Zhan X., Goldfarb M. (1986). Growth factor requirements of oncogene-transformed NIH 3T3 and BALB/c 3T3 cells cultured in defined media.. Mol Cell Biol.

